# HER2-Positive Breast Cancer—Current Treatment Management and New Therapeutic Methods for Brain Metastasis

**DOI:** 10.3390/biomedicines13051153

**Published:** 2025-05-09

**Authors:** Hanna Miski, Kamila Krupa, Michał Piotr Budzik, Andrzej Deptała, Anna Badowska-Kozakiewicz

**Affiliations:** 1 Students’ Scientific Organization of Cancer Cell Biology, Department of Oncology Propaedeutics, Medical University of Warsaw, 01-445 Warsaw, Poland; s090695@student.wum.edu.pl (H.M.); kamila.krupa@student.wum.edu.pl (K.K.); 2 Department of Oncology Propaedeutics, Medical University of Warsaw, 01-445 Warsaw, Poland; andrzej.deptala@wum.edu.pl (A.D.); anna.badowska-kozakiewicz@wum.edu.pl (A.B.-K.)

**Keywords:** HER2-positive breast cancer, brain metastasis, trastuzumab, CNS, trastuzumab emtansine, metastatic breast cancer

## Abstract

**Background**: Breast cancer can be classified based on the immunohistochemistry (IHC) phenotypes, defined by the presence or absence of the main IHC markers. IHC phenotyping is important as it determines the prognosis and guides treatment. For example, human epidermal growth factor receptor 2 (HER2) overexpression, which triggers cell growth and division, is observed in HER2-positive breast cancer. **Methods**: The standard treatment is based on trastuzumab plus pertuzumab in combination with taxane chemotherapy. The possibility of developing metastases depends on those phenotypes. Approximately 25–50% of patients with HER2-positive breast cancer experience brain metastases. This aspect is especially important, as 20% of those patients die as a result. **Results**: Through the years, many advanced therapies have been introduced to treat brain metastases, including whole brain radiotherapy, stereotactic radiosurgery, and a tyrosine kinase inhibitor (TKI), neratinib. Nonetheless, this still remains a therapeutic challenge. **Conclusions**: In this review, we focus on the treatment and efficiency of therapies targeting HER2-positive breast cancer, mainly concentrating on the current and newly developed treatment options for brain metastases, such as trastuzumab deruxtecan and tucatinib.

## 1. Introduction

HER2 is a receptor tyrosine kinase (RTK) belonging to the epidermal growth factor receptor (EGFR) family [1]. This family comprises four receptors sharing five structural domains: a hydrophobic transmembrane domain, a glycosylated extracellular domain, a juxtamembrane segment, a tyrosine kinase domain, and a tyrosine-rich C-terminal tail [2]. The extracellular region consists of four subdomains—domains I and III (ligand binding) and domains II and IV (intramolecular interactions) [3]. Those family members are activated by ligands in an autocrine, paracrine, or juxtacrine manner [4]. HER2 is unique because it has no known ligand and functions through ligand-induced heterodimerization, particularly with HER3. HER2/HER3 heterodimers are frequently observed in breast cancer (BC) and are critical for activating downstream signaling pathways such as Ras/Raf/MAPK, which drive cell proliferation [5]. Although normal cells express up to 100,000 EGFRs, cancer cells can express up to 2 million, and HER2 overexpression is associated with tumor aggressiveness and resistance to chemotherapy [1].

When cancer cells from the primary tumor transfer to the brain through the bloodstream and proliferate, a metastatic brain tumor is developed. Approximately 25–50% of women with HER+ metastatic breast cancer (MBC) develop brain metastases (BrM) [6], which significantly reduces survival—20% of BC patients die from BrM [7]. Despite this, HER2+ MBC patients often have better outcomes than other subtypes due to effective anti-HER2 therapies [8]. Studies have detected several potential reasons explaining the high risk of BrM in HER2+ BC. One of the factors that drives BC BrM is the interaction and signaling of HER2 and EGFR/HER3. BrM overexpresses HER2 and HER3 and the brain microenvironment consists of HER family ligands that trigger dimerization and activation of receptors in brain metastatic cells. The HER2/HER3 dimmer seems particularly important as it may cause BrM by releasing matrix metalloproteases that can corrupt the BBB [6,9]. Previously, patients with baseline BrM were excluded from clinical trials. Lapatinib was among the first drugs explicitly studied in these patients [10]. Current treatment strategies include surgery, whole-brain radiotherapy, stereotactic radiosurgery, chemotherapy, and anti-HER2 agents such as trastuzumab, pertuzumab, trastuzumab emtansine, lapatinib, and neratinib [11]. In some cases, like leptomeningeal metastases, treatment decisions rely heavily on expert consensus [12]. Median survival without treatment for BrM is about 1 month [13]. Studies have shown that long-term BC BrM survivors tend to be younger, with fewer brain lesions, higher rates of complete response to neoadjuvant chemotherapy, less frequent extracranial or leptomeningeal spread, and asymptomatic BrM at the time of diagnosis [14].

## 2. Mechanisms and Clinical Implications of HER2 Overexpression

**Figure 1:**
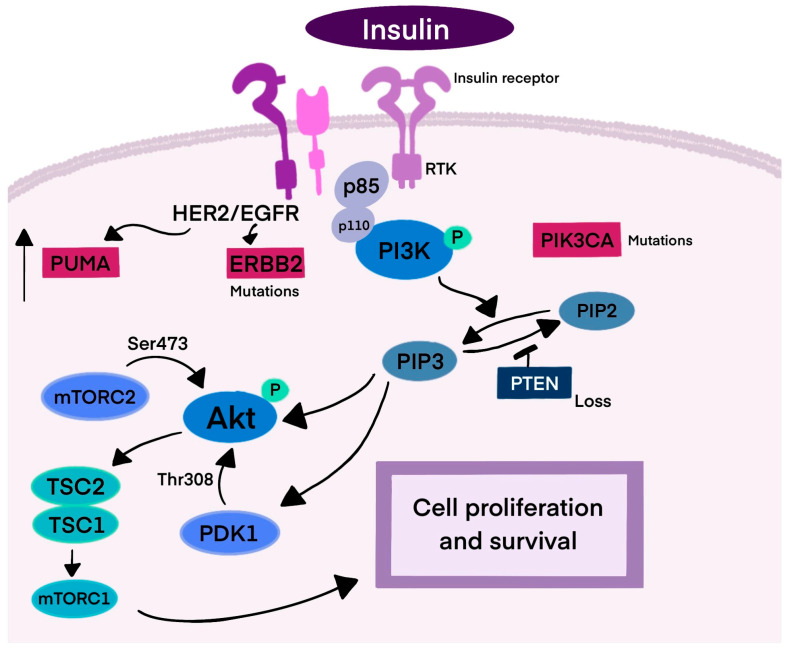
Mechanism of HER2 signaling. This figure represents the signaling pathway leading to cancer cell survival and proliferation. Breast cancer is one of three cancers with the highest incidence of PIK3CA mutations. PTEN negatively regulates the PI3K cascade, inhibiting cell proliferation. HER2 receptors also interact with PUMA, resulting in its degradation and survival of the cancer cell. Abbreviations: RTK—receptor tyrosine kinase; PI3K—phosphoinositide 3-kinase; p85/p110—subunits of PI3K; PIP3—phosphatidylinositol (3,4,5)-trisphosphate; PIP2—phosphatidylinositol 4,5-bisphosphate; Akt—protein kinase B; mTORC2—mTOR complex 2; PDK1—3-phosphoinositide dependent protein kinase-1; PUMA—p53-upregulated modulator of apoptosis; TSC2—tuberous sclerosis complex 2; TSC1—tuberous sclerosis complex 1; ERBB2—human epidermal growth factor receptor 2; PIK3CA—phosphatidylinositol-4,5-bisphosphate 3-kinase catalytic subunit alpha; PTEN—phosphatase and tensin homolog deleted on chromosome ten.

Ligand binding to a receptor tyrosine kinase (RTK), such as insulin, activates phosphoinositide 3-kinase (PI3K) [15]. PI3K consists of two subunits: p85, which binds to EGFR tyrosine phosphorylated sites, and p110, which produces PIP3, leading to Akt kinase activation. PIP3 binds AKT and PDK1 to the plasma membrane, and AKT is phosphorylated by mTORC2 and PDK1, promoting cell proliferation. Mutations in PIK3CA, the catalytic subunit of PI3K, are common in BC [5,16,17,18,19,20]. PTEN, codifying a lipid phosphatase, negatively regulates this pathway, controlling cell survival and growth. Additionally, mutations in TP53 and enhanced HER2 activation or dimerization can contribute to HER2+ BC, with HER2 interacting with p53 upregulated modulator of apoptosis (PUMA) to promote cancer cell survival (Figure 1) [21,22,23].

HER2 overexpression is primarily caused by HER2 gene amplification. In HER2-overexpressing and amplified BC cells, HER2 mRNA levels are 4- to 8-fold and 64-128-fold higher than expected based on gene copy numbers. HER2 expression is regulated by several transcription factors, including TFAP2, Sp1, YY1, ETS, YB-1, and EGR2 (positive regulation), and MYB, FOXP3, GATA4, PEA3, MBP-1, NOTCH, and RBP-Jk (negative regulation) [15]. Another mechanism controlling gene expression is hypermethylation, particularly in the promoter region. Methylation can inhibit transcription by blocking transcription factor-binding or attracting methyl-CpG-binding proteins, leading to the silencing of tumor suppressor genes and uncontrolled cell proliferation. Hypermethylation of the promoter of tumor suppressor genes (TSGs) induced by transforming growth factor beta-induced (GFBI) can be the reason for primary or acquired resistance to trastuzumab [24,25].

Wdr5, a component of the H3K4 methyltransferase complex, is involved in increased ErbB2 expression in BC. It is essential for H3K4me3 enrichment of the ErbB2 promoter, and this mark is required for acquiring the H3K4ac mark in ErbB2-amplified tumors. Targeting Wdr5 in chemotherapy or trastuzumab reduces ErbB2 overexpression and inhibits cancer cell growth [26,27]. Additionally, microRNAs (miRNAs)—small oligonucleotides of ~20 nucleotides—play a role in cancer progression. Onco-miRNAs promote tumor onset and proliferation, while metasta-miRNAs impact metastasis, making them potential prognostic markers or therapeutic targets in BC [28,29].

p95 is a form of HER2 lacking the extracellular domain, which acts as an inhibitor until ligand binding activates it. Because p95 lacks this domain, it is resistant to trastuzumab, as it is not recognized by antibodies targeting the extracellular region [30].

Normal tissues have low levels of HER2 [31], but EGFRs are found in human skin, particularly in basal keratinocytes located in the epidermis and epidermal appendages, hair follicles, sebaceous, and sweat glands. It is also expressed during fetal skin development and in fibroblasts and endothelial cells, supporting tissue integrity [32]. EGFRs are essential for normal function in the placenta, reproductive system, and respiratory tract [33]. The ErbB family is involved in organ growth, including the mammary gland and central nervous system [34], and EGFR mutations are common in glioblastoma, promoting tumor growth [35].

The neuregulin (NRG)/ErbB signaling pathway is vital for heart development, including trabeculae formation, angiogenesis, and valve formation. Myocytes lacking ErbB2 are more susceptible to anthracycline-induced toxicity and dilated cardiomyopathy [36]. HER2 gene mutations, though less common than amplifications, can alter the structure and function of the protein and have been identified in cancers such as breast, gastric, lung, and colorectal [37]. HER2 amplification is present in 3–5% of colorectal cancers and is associated with more aggressive tumor behavior and higher recurrence rates [38].

HER2 receptors are present during Schwann cell development. Schwann cells, the most abundant glial cells of the peripheral nervous system, play an essential role in the development and function of nerves [39]. The ErbB2/neuregulin-1 (Nrg1) signal indicates proliferation of the Schwann cell precursors, stimulates their migration along the axon, and controls myelination. It has also been proved that the disruption of ErbB2 causes hypomyelination. Both of those molecules are also expressed in adulthood, which suggests their contribution to mature Schwann cells’ functioning [40].

The expression levels of estrogen receptor (ER), progesterone receptor (PR), and HER2 are useful response predictors to hormonal therapy and allow the selection of appropriate treatment (Table 1) [41]. HER2+ cancers represent 15–20% of all BC cases. It is classified as aggressive and often associated with metastasis and poor prognosis [42]. However, the triple-negative BC has the worst prognosis among small tumors (<1 cm) [43]. There are various prognostic biomarkers for HER2+ BC, but none to define which HER2-targeted therapy will be the most effective [44]. Studies have shown that some factors are correlated with HER2 overexpression in BC. Those factors are age over 50, higher histological grade, and higher T stage [45]. Much data shows that biological behavior, clinical features, therapeutic response, and prognosis of HER2+ BC depend on hormone receptor (HR) status. In fact, tumors considered HR+/HER2+ have shown a lower pathological complete response rate than HR-/HER2+. Moreover, HR-/HER2+ BC has a higher risk of recurrence in the first 5 years than HR+/HER2+ BC [46].

## 3. Monoclonal Antibodies in HER2-Positive Tumors

Currently, trastuzumab, pertuzumab, and margetuximab are approved by the US Food and Drug Administration (FDA) and/or the European Medicines Agency (EMA) in HER2+ BC treatment. Trastuzumab (Herceptin) binds to the extracellular domain IV of HER2, inhibits the ligand-independent HER2/HER3/PI3K signaling complex, enhances antibody-dependent cellular cytotoxicity (ADCC), and ultimately leads to cancer cell death [47,48]. In the Vogel et al. study (2002), the objective response rate (ORR) was 26% in patients with HER2-overexpressing MBC, and trastuzumab was well tolerated as a first-line therapy. The most common adverse events (AE) were chills, asthenia, fever, pain, and nausea [49]. It was the first specific anti-HER2 monoclonal antibody available for HER2+ BC patients. However, some of them (approximately 35%) exhibit de novo resistance, or they may be developed after trastuzumab therapy (about 70%), resulting in disease progression [50]. Moreover, cardiotoxicity is the main concern during therapy [51].

Pertuzumab inhibits HER2/HER3 dimerization and signaling by targeting the extracellular domain II of HER2 [52,53]. In the phase III CLEOPATRA study (NCT00567190), the combination of pertuzumab, trastuzumab, and docetaxel as first-line treatment for HER2+ MBC significantly extended progression-free survival (PFS) compared to placebo plus trastuzumab plus docetaxel (18.5 vs. 12.4 months; *p* < 0.001) without increasing cardiac AEs [54]. A median follow-up of 50 months showed significant improvement in overall survival (OS) (56.5 months vs. 40.8 months; *p* < 0.001) and sustained PFS improvement in the pertuzumab plus trastuzumab plus docetaxel group [55]. In the phase III APHINITY trial (NCT01358877), adding pertuzumab to trastuzumab and chemotherapy improved the rates of invasive disease-free survival (iDFS) among patients with HER2+ early breast cancer (EBC) [56]. Updated 8-year iDFS data for the node-positive population indicated a 4.9% absolute improvement in favor of pertuzumab; however, pertuzumab did not improve OS rates (92.7% in the pertuzumab versus 92.0% in the placebo group; *p* = 0.078). Additionally, the node-negative cohort responded well without pertuzumab in the combination at this third interim analysis [57]. The phase III PEONY trial NCT02586025 showed statistically significant improvement in the total pathologic complete response rate for pertuzumab plus trastuzumab plus docetaxel combination as a neoadjuvant treatment of HER2+ early or locally advanced BC in Asian patients [58]. The final 5-year analysis exhibits meaningful benefit in event-free survival (EFS) (84.8% vs. 73.7%; HR 0.53) and disease-free survival (DFS) (86.0% vs. 75.0%; HR 0.52) for the combination with pertuzumab. A positive trend in OS was also noted (93.9% vs. 90.0%; HR 0.53). Subgroup analyses highlighted better outcomes in patients with high-risk features, such as lymph node-positive or HR-negative disease and in the higher HER2 mRNA subgroup, and worse in a subgroup of patients with PIK3CA mutations [59].

Margetuximab (MGAH22) is a novel monoclonal antibody derivative of trastuzumab that binds to the same epitope of the HER2 receptor with similar affinity and antiproliferative effects as trastuzumab. However, its IgG1 Fc region is engineered to have greater affinity for the stimulatory CD16A FcγRIIIA receptor on natural killer (NK) cells and reduced affinity for the inhibitory CD32B FcγRIIB receptor on immune effector cells. These modifications enhance the antibody-dependent cellular cytotoxicity (ADCC) process, improving the host’s immunological recognition of cancer cells [60]. Following the preliminary results of the SOPHIA trial (NCT02492711), it received Food and Drug Administration (FDA) approval in 2020 for patients with locally advanced or metastatic HER2+ BC who have previously undergone at least two anti-HER2 treatments, including one for metastatic disease [61]. At data cutoff, with a median follow-up of 20.2 months, the median OS in the intention-to-treat (ITT) patients did not differ significantly between the two treatment groups (21.6 vs. 21.9 months with margetuximab and trastuzumab, respectively; *p* = 0.620). In patients who carried the CD16A-158F low-affinity allele, margetuximab prolonged the OS by 2.5 months compared with trastuzumab (23.3 vs. 20.8 months). Similarly, among CD16A-158FF patients, the OS was extended by 4.4 months (23.6 vs. 19.2 months). For CD16A-158FV patients, median OS was not significantly different, while CD16A-158VV patients had better outcomes with trastuzumab (21.3 vs. 22.0 months; 22.0 vs. 31.1 months, respectively). The survival advantage for margetuximab plus chemotherapy was not observed in the final analysis of OS; however, the results suggest that the CD16A genotype may be a significant predictor of response to margetuximab plus trastuzumab treatment [62].

The phase II MARGOT trial (NCT04425018) evaluates how well participants with stage II-III HER2+ BC respond to pre-operative treatment using one of two combinations: paclitaxel plus pertuzumab plus margetuximab or paclitaxel plus pertuzumab plus trastuzumab [63].

Antibody–drug conjugates are targeted therapies for cancer treatment that combine a monoclonal antibody specific to a tumor-associated antigen with a cytotoxic drug, combined by a chemical linker [64]. ADCs deliver cytotoxic agents directly to cancer cells, minimizing damage to normal tissues. The ADC internalizes when it binds to the target antigen on the cancer cell’s surface, releasing the cytotoxic agent inside the cell and resulting in cell death. This strategy enables the delivery of greater amounts of the cytotoxic drug to cancer cells, reducing systemic toxicity [Lim D., 1 December 2022 #174] [65]. As of now, there are fifteen ADCs approved by the FDA, of which two (trastuzumab emtansine and trastuzumab deruxtecan) are used in treating HER2+ BC [66].

Monoclonal antibodies in prodrug conjugates, immunostimulatory conjugates, engineered toxins, and radiopharmaceutical conjugates are also being used to explore new possibilities for blocking HER2 signaling [67]. In preclinical studies, one of the novel ADCs, DHES0815A, demonstrated efficacy and tolerability. However, in the phase I study (NCT03451162), persistent, non-resolving dermal, ocular, and pulmonary adverse events led to early termination of this trial, even though the drug exhibited early signs of anti-tumor activity [68]. Another interesting ADC drug targeting HER2 is MRG002, which has reduced antibody-dependent cell-mediated cytotoxicity in comparison to trastuzumab and is covalently bonded by a valine-citrulline (vc) protease-cleavable linker to monomethyl auristatin E (MMAE). All those drugs are being assessed in clinical trials (NCT05018676, NCT02952729, NCT04742153) [69,70]. Recently, D. Lim and colleagues created an ADC based on trastuzumab coupled with a powerful payload of the Duocarmycin family (US11938115B2), which showed increased cytotoxicity against cancer cells that were HER2+ [71].

Treatment with ADCs such as trastuzumab emtansine has been associated with the development of resistance, highlighting the need to find another effective drug. The ARX788 was created using a unique non-cleavable drug linker and cytotoxic tubulin inhibitor (AS269), and XMT-1522 includes a cysteine linkage and a humanized IgG1 anti-HER2 antibody (HT-19) [70,72,73]. Both drugs showed effectiveness in HER2+ resistance to trastuzumab emtansine, so that they could be a possible treatment option in patients with advanced HER2+ BC [72,73]. There has been enormous progress with ADCs, and their modifications may significantly improve the treatment of HER2+ BC patients, not only in the future. However, more research is needed to assess their efficacy and activity fully.

In addition to monoclonal antibodies, several small TKIs such as lapatinib, neratinib, and tucatinib have been developed to target the intracellular domain of HER2 [74]. These agents are relevant in the treatment of HER2+ BC with CNS involvement or resistance to antibody-based therapy. The usage of those drugs, especially in HER2+ BC with BrM, will be discussed in further sections.

## 4. Recommendations for the Treatment of HER2-Positive BC

Current guidelines from the National Comprehensive Cancer Network^®^ (NCCN^®^), the European Society for Medical Oncology (ESMO), and the American Society of Clinical Oncology (ASCO^®^) recommend anti-HER2 monoclonal antibody treatment with trastuzumab plus pertuzumab in combination with taxane chemotherapy as the standard of care for first-line systemic therapy in HER2+ MBC [75,76,77,78]. For patients with HR+/HER2+ BC who are ineligible for chemotherapy, a chemotherapy-free regimen using anti-HER2 agents and hormone therapy (e.g., trastuzumab with or without pertuzumab combined with endocrine therapy—ET) may be considered. The TAnDEM and eLEcTRA studies have shown that adding trastuzumab to an aromatase inhibitor (AI), anastrozole and letrozole, respectively, prolonged PFS, though no clear benefit in OS was observed [79,80]. The CLEOPATRA trial did not allow the use of ET before progression for those with HR co-expression, so the purpose of the phase III SYSUCC-002 trial (NCT01950182) was to define if trastuzumab plus ET is noninferior to trastuzumab plus chemotherapy in patients with HR+/HER2+ MBC. The median PFS was 19.2 months in the ET group and 14.8 months in the chemotherapy group. Additionally, patients in the ET group had a significantly lower prevalence of AEs of grade 3 to 4 [81].

The phase II PERTAIN trial (NCT01491737) demonstrated the benefit of dual HER2 blockade (trastuzumab plus pertuzumab) with an AI. Median PFS was 18.89 months in the pertuzumab plus trastuzumab arm and 15.8 months in the trastuzumab arm (*p* = 0.007) [82]. Additionally, in the phase III ALTERNATIVE trial (NCT01160211), dual HER2 blockade with lapatinib plus trastuzumab plus AI showed superior PFS benefit versus trastuzumab plus AI in patients with HR+/HER2+ MBC (11 vs. 5.6 months; *p* = 0.0063) [83].

In 2007, the FDA first approved lapatinib, a reversible dual HER1/HER2 kinase inhibitor, in combination with capecitabine for MBC that has advanced previous treatments [84]. However, its common side effect, diarrhea [84], limits some clinical use, so novel treatment strategies are being discovered.

T-DM1 (trastuzumab emtansine), an ADC, is one of the choices for second-line treatment, and it was the first ADC approved by the FDA in 2013 for treating HER2+ MBC [85]. In the phase III EMILIA trial (NCT00829166), it showed improvement in PFS and OS compared with lapatinib plus capecitabine by 3.2 months (*p* < 0.0001) and 5.8 months (*p* = 0.0006), respectively [86]. The Phase III TH3RESA trial (NCT01419197) also demonstrated the benefit of T-DM1 in third-line treatment, demonstrating improved PFS and OS. The median PFS was significantly improved with T-DM1 compared with the physician’s choice treatment (6.2 vs. 3.3 months; *p* < 0.0001) [87]. The final OS analysis showed significant improvement for the T-DM1 group (22.7 vs. 15.8 months; *p* = 0.0007) [88]. In 2019, based on the results of the KATHERINE trial, T-DM1 was also approved for adjuvant treatment of HER2+ EBC with residual invasive disease following neoadjuvant trastuzumab and chemotherapy [89].

Trastuzumab deruxtecan (T-DXd) is now the preferred second-line treatment for patients with HER2+ MBC who have received prior anti-HER2 therapy [75]. This ADC is composed of trastuzumab linked to a topoisomerase I inhibitor [90]. This recommendation is based on the results from the phase 3 DESTINY-Breast03 trial (NCT03529110), in which trastuzumab deruxtecan provided significantly longer PFS than T-DM1 regardless of HR status [91]. The percentage of those who were alive without disease progression at 12 months was 75.8% in trastuzumab deruxtecan and 34.1% in the trastuzumab emtansine group (*p* < 0.001). The trastuzumab deruxtecan group also showed a higher overall response (79.7% vs. 34.2%). While the percentage of AE of grade 3 or higher was comparable in the two groups, the rate of trial treatment discontinuation due to AE was greater in the trastuzumab deruxtecan group [91]. According to a network meta-analysis of anti-HER2 treatments, T-DM1 is less effective than T-DXd and pyrotinib plus capecitabine in terms of PFS and ORR, but more effective than combinations of lapatinib plus capecitabine, lapatinib plus trastuzumab, and neratinib plus capecitabine. Compared to T-DXd, T-DM1 has a better safety profile, with fewer serious AEs and lower discontinuation rates [92]. Based on phase II DESTINY-Breast01 (NCT03248492), fam-trastuzumab deruxtecan-nxki received FDA accelerated approval in 2019 for unresectable or metastatic HER2+ BC, in patients who have received two or more prior anti-HER therapies [93]. The ORR from updated results was 62%, the median duration of response (DoR) was 18.2 months, and PFS was 19.4 months [94]. T-DXd can also target tumor cells with low expression of HER2 and can deliver its potent cytotoxic payload [90,95]. Phase 3 trial DESTINY-Breast04 (NCT03734029) showed that T-DXd resulted in significantly longer PFS and OS than the physician’s choice of chemotherapy in patients with HER2-low MBC [96]. These findings suggest a novel therapeutic strategy for patients previously diagnosed with HER2-negative BC, which may now be classified as HER2-low [97].

Tucatinib, neratinib, and margetuximab are used following the progression of pertuzumab, T-DXd, and T-DM1 [75]. Tucatinib, in combination with trastuzumab and capecitabine, demonstrated efficacy in heavily pretreated patients with HER2+ MBC in the phase II HER2CLIMB study (NCT02614794). The median PFS at one year was 7.8 months in the tucatinib combination group compared to 5.6 months in the placebo group, and the median OS was 21.9 months vs. 17.4 months, respectively. However, grade 3 or higher AE-like diarrhea or elevated aminotransferase levels were more common in the tucatinib group [98]. Another TKI—neratinib, inhibiting HER1, HER2, and HER4, received FDA approval in 2017 for extended adjuvant treatment of early-stage HER2+ BC following adjuvant trastuzumab, based on the phase III ExteNET trial (NCT00878709) [99,100]. In this trial, the iDFS after 5.2 years was higher in the neratinib group versus placebo (90.2% vs. 87.7%; *p* = 0.008). Moreover, the neratinib group had significantly fewer iDFS events (116 vs. 163 events; *p* = 0.0083) [101]. Final efficacy results from the phase III ExteNET trial after 5 years of follow-up showed that neratinib was associated with an absolute increase in iDFS of 5.1% compared with placebo in patients with HR+/HER2+ EBC who initiated treatment ≤ 1 year post-trastuzumab. Moreover, after 8 years, there was an improvement in OS with an absolute increase of 2.1% in favor of neratinib, and after 5 years, DFS increased by 4.7% in favor of neratinib. The advantage was negligible for the patients who started neratinib therapy more than a year after finishing trastuzumab (1.3% in iDFS at 5 years) [102]. In the NALA study (NCT01808573), neratinib in combination with capecitabine improved PFS (median PFS 8.8 vs. 6.6 months), but not OS, in comparison to lapatinib and capecitabine. This led to FDA approval in 2020 for neratinib plus capecitabine in patients with HER2+ advanced breast cancer (ABC) or MBC who have received two or more prior anti-HER2 regimens in the metastatic setting [103,104].

Pyrotinib, a pan-HER TKI, showed improved PFS when combined with capecitabine compared to lapatinib plus capecitabine in the phase III PHOEBE study (NCT03080805) in HER2+ MBC patients previously treated with trastuzumab and chemotherapy. The median PFS was 12.5 months for pyrotinib plus capecitabine, compared with 6.8 months for lapatinib plus capecitabine (*p* < 0.001) (Table 2) [105].

New and promising therapies are focused on novel ADCs, such as trastuzumab duocarmazine (SYD985) and PF-06804103, combinations of immune checkpoint inhibitors with HER2-targeted therapies, and combinations of CDK4/6 inhibitors, especially in combination with hormonal therapy and anti-HER2. Data from the phase III MUKDEN 07 trial (NCT05635487), presented at the 2024 ESMO Congress, showed that SHR-A1811, a novel anti-HER2 ADC, in combination with pyrotinib, led to antitumor activity as neoadjuvant therapy for HER2+ BC, with an ORR of 89.7% [106].

## 5. Metastases to the Brain—Current Therapeutic Methods

### 5.1. Onset of Brain Metastases in HER2+ BC Patients

**Figure 2:**
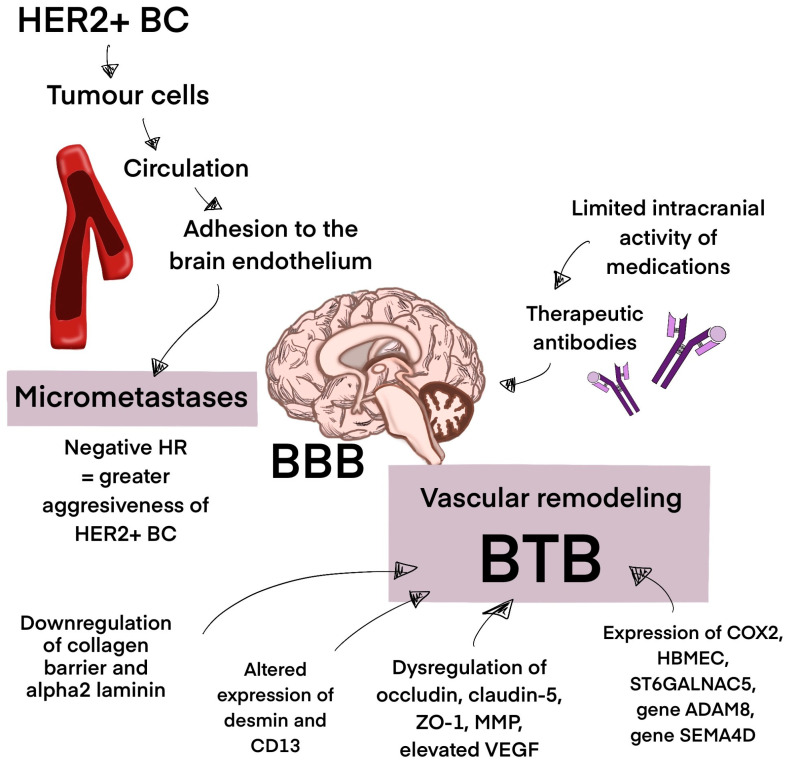
The process of brain metastasis development. This figure represents the process of brain metastasis development in HER+ BC. The BBB in the process of vascular remodeling turns into a porous BTB, supporting micrometastases development. Micrometastases eventually occupy the brain. Abbreviations: BBB—blood–brain barrier; BTB—blood–tumor barrier; HR—hormone receptor; HER2+ BC—HER2-positive breast cancer, ZO-1—zonula occludens-1; MMP—matrix metalloproteinases; VEGF—Vascular Endothelial Growth Factor; COX-2—cyclooxygenase-2; HBMEC—human brain microvascular endothelial cells; ST6GALNAC5—a2,6-sialyltransferase.

The high incidence of BrM in HER2+ BC patients can be attributed to the limited intracranial activity of medications and the HER+ tumor cells’ susceptibility to therapies that increase patient survival [107]. HER+ BC also has a biological propensity to metastasize to the brain [108]. Therapeutic antibodies such as trastuzumab have a poor ability to cross the BBB, which limits their efficacy in patients with established brain metastases; however, in patients with advanced stages of HER+ BC, they have been shown to postpone the onset of symptomatic brain metastases [107,109,110]. Moreover, HER+ BC is considered aggressive, and negative hormone receptors are a risk factor for BrM. The process of BrM begins when breast epithelial cancer cells at the initial location start to exhibit mesenchymal traits. The tumor extracellular matrix (ECM) breaks down due to the ongoing generation of primary tumor neovascularization. Tumor cells enter the circulation system, developing into circulating tumor cells and, after adhering to brain endothelial cells and navigating the cerebral BBB MBC cells, develop micrometastases, eventually occupying the brain [111,112,113,114]. BC cells appear to operate paracellularly to cross the BBB, squeezing past endothelial cells by intercellular connection disruption. Certain modifications are performed to improve the traverse of cancer cells through the BBB. Through vascular remodeling of pre-existing brain arteries, including downregulating basement membrane collagen part IV and laminin α2, BC cells assemble along brain endothelial cells, transforming the previously intact BBB into a porous blood–tumor barrier (BTB). BC BrM has been found to have a shift in pericyte phenotypes that involve altered expression of desmin and CD13, which impacts the barrier’s permeability. Dysregulation of occludin, claudin-5, zonula occludens-1 (ZO-1), and matrix metalloproteinases (MMPs), as well as elevated levels of Vascular Endothelial Growth Factor (VEGF), support the penetration of the BBB. Moreover, the expression of cyclooxygenase-2 (COX-2), human brain microvascular endothelial cells (HBMEC), and a2,6-sialyltransferase (ST6GALNAC5), as well as genes such as ADAM8 and SEMA4D, has been shown to mediate passage across the BBB [115,116,117,118,119,120]. However, the penetration of the BBB by therapeutic drugs remains a challenge. Loss of PTEN expression and the activation of the PI3K-AKT-mTOR pathway play a significant role in drug resistance mechanisms. Activation of the neuregulin-HER3 axis contributes to this topic (Figure 2) [121].

### 5.2. Whole Brain Radiotherapy (WBRT)

Whole brain radiotherapy (WBRT) has been used as a standard treatment for BC BrM, but new research suggests that it may be more beneficial for instances with five to ten lesions spread across multiple disease sites. However, its high efficiency relieves symptoms in 70% of patients, which can be used as a palliative treatment [122]. A total of 30 Gray (Gy) in 10 fractions over 2 weeks or, alternatively, 20 Gy in 5 fractions is the standard dose given to patients for BrM treatment. Sometimes the dose of 40 Gy in 20 fractions is used to reduce the risk of late radiation-induced encephalopathy with neurocognitive disturbances. However, the local control using only WBRT is estimated to be around 62% [123,124]. This type of treatment also comes with long-term side effects. One of those is the decline of neurocognitive functions, so many strategies, such as intensity-modulated radiotherapy (IMRT) or drugs like memantine or donepezil, have been introduced. Hippocampal-avoidance WBRT (HAWBRT), a conformal WBRT delivered by IMRT, is used to lower the radiation dose to the bilateral hippocampi, which is significant in memory preservation [125]. It has been reported that 35–52% of patients experience a decline in cognitive function within 3–6 months after WBRT. The QUARTZ trial demonstrated no significant difference in median OS or quality of life between patients receiving WBRT and supportive care, and the group receiving just supportive care [126]. Median OS after WBRT alone is between 3 and 6 months [127].

Studies performed in 2010 by Niwińska et al. showed that the median survival after WBRT without further treatment was 2 months for HR+/HER2+ and 4 months for HER2+ ER/PR(-) patients [13].

In a studies performed from 1999 to 2012 in Stockholm, Sweden, the median survival after WBRT was 2.9 months; moreover, one in four patients could not be discharged from the hospital following this procedure [118]. Generally, WBRT is associated with memory disturbance, difficulty with complex problem solving, ataxia, and urinary incontinence; however, it is shown to reduce brain recurrences and deaths from neurologic causes [128]. The treatment of brain metastases with WBRT is also associated with the risk of radiation necrosis [123].

### 5.3. Stereotactic Radiosurgery

The American Society for Radiation Oncology guidelines state that stereotactic radiosurgery (SRS) should be a treatment considered for patients with brain metastases of up to four lesions, good performance status, and well-controlled extracranial disease [129]. This method reduces the cognitive side effects frequently linked to WBRT, minimizes harm to nearby healthy tissues, and provides a highly conformal radiation dose while precisely targeting metastatic lesions. Its local control rates are 65% to 86% at one year for lesions up to 1.5 cm [130]. The Radiation Therapy Oncology Group advises stereotactic radiosurgery for brain metastases with a maximum diameter of 4.0 cm. The maximum tolerated dose is 24 Gy for brain metastases up to 2.0 cm in diameter, 18 Gy for 2.1–3.0 cm, and 15 Gy for 3.1 to 4.0 cm [131].

A study by Fabian et al. assessed the efficacy of metastasis-directed SRT in BC patients. A total of 66% of patients at the time of SRT had no more than five metastases. A total of 73% of those lesions were intracranial and 27% were extracranial. A total of 33% of patients had been diagnosed with HER2+ BC. After 12 and 24 months, the cumulative incidence of local recurrence across metastatic sites was 13% and 20%, respectively. Intracranial lesions had higher recurrence rates after 12 months (15% vs. 5.8%) and after 24 months (25% vs. 7.3%). The median PFS for all patients was 8.7 months. However, patients receiving SRT to extracranial metastases had longer PFS than those receiving it to intracranial metastases (13.8 months vs. 7.3 months). Moreover, patients treated for extracranial lesions had a higher OS (44.8 months vs. 18.5 months) [132].

Stafinski et al. observed no difference in survival between WBRT+ SRS and WBRT alone in patients with numerous metastases. Nonetheless, patients with a single metastasis found a statistically significant survival benefit favoring WBRT+ SRS. Furthermore, regardless of the number of metastases, the WBRT+ SRS therapy arm had noticeably greater local tumor control rates at 24 months [133]. When treating multiple targets with SRS, there is a risk of radiation necrosis. SRS is usually performed using the Gamma Knife, and rates of radiation necrosis on imaging in patients treated with it vary from 0 to 12.2% [134]. SRS is most effective for small to medium metastases. Larger lesions pose challenges due to limiting radiation exposure to surrounding healthy tissues. After SRS, lesions can take weeks or months to respond or shrink [135]. For patients with longer survival times, SRS can pose a risk of neurocognitive decline [130].

### 5.4. Trastuzumab

Many chemotherapeutic drugs and HER2-targeted treatments may not reach adequate therapeutic levels to eliminate metastatic cells because they cannot pass across the BBB or are pumped out of the CNS by P-glycoproteins found in the BBB.

BrM occurs because cancer cells can escape the cytotoxic efficacy of systemic therapy. The BBB can be damaged by a growing tumor, cranial surgery, or brain radiotherapy. Studies show improved survival after the development of BrM in patients treated with chemotherapy with trastuzumab [127]. Stemmler et al. discovered a 420-fold lower concentration of trastuzumab in cerebrospinal fluid than in serum in patients receiving this drug before radiotherapy [109].

It has been noted that when individuals with MBC receive trastuzumab before being diagnosed with brain metastases, the period before BrM occurs is doubled compared to those who do not receive trastuzumab before BrM. The OS for patients receiving trastuzumab after BrM was 13.6 months compared with 5.5 months for patients never receiving this drug. However, receiving it after BrM elongates the survival time compared to patients treated with conservative treatment or other systemic chemotherapy [136].

AEs linked to trastuzumab alone are composed of unspecified pain, asthenia, nasopharyngitis, skin disorders (primary rush), dyspepsia, paresthesia, infections, increased lacrimation, diarrhea, myalgia, edema, fever, nose bleeds, cardiac events, insomnia, cough, back pain, dyspnea, chills, dizziness, hypertension, congestive heart failure, increased aspartate aminotransferase levels, gastrointestinal issues, and dehydration [137]. It is important to note that the use of trastuzumab can be associated with cardiac AEs. Trastuzumab-related cardiotoxicity is observed in 15–20% of patients, and less than 5% of patients experience heart failure. Moreover, 40–45% of patients show decreases in cardiac function of 10% [138].

### 5.5. Pertuzumab

In the PATRICIA study, patients with progressed BrM after radiotherapy received a standard dose (initial dose of 840 mg, followed by a 420 mg maintenance dose every 3 weeks) of pertuzumab with a high dose of trastuzumab (6 mg/kg weekly) until central nervous system (CNS) progression or unacceptable toxicity. A total of 70.0% of patients received prior WBRT at the first diagnosis of BrM, and 54.5% received SRS at the time of progression. Moreover, the median time from the last radiotherapy to study entry was 18.6 months. As a result of this trial, the median CNS-PFS was 4.6 months, and the median OS was 27.2 months. The ORR was 11%. The most frequently detected AEs were diarrhea, fatigue, nausea, vomiting, constipation, dizziness, headache, and insomnia [139,140].

The CLEOPATRA phase III trial was constructed using pertuzumab, trastuzumab, and paclitaxel. It was discovered that there was no discernible difference in the incidence of brain metastases between the therapy groups—the pertuzumab arm and the placebo arm—nonetheless, pertuzumab increased the time to development of BrM [12,141].

A study conducted in 2023 compared the efficacy and safety of pyrotinib plus trastuzumab (PyroH) and pertuzumab plus trastuzumab (HP). A total of 48.3% of patients received PyroH, and the rest received HP. A follow-up conducted 16 months after this study showed that 105 out of 161 patients in the PyroH group and 80 out of 172 patients in the HP group experienced disease progression. Patients with brain metastases from the PyroH cohort showed a longer median PFS than patients from the HP cohort (9.03 vs. 8.15 months). The median OS was not reached. The pyrotinib and trastuzumab combination is more effective without taxane and is comparable in second-line or later treatment as well as during BrM [142].

### 5.6. Trastuzumab Emtansine

Another humanized monoclonal antibody used in MBC treatment is trastuzumab emtansine, also called ado-trastuzumab emtansine (T-DM1). It is an ADC composed of the antibody to the HER2 receptor conjugated via maleimidomethyl cyclohexane-1-carboxylate (MCC) to the cytotoxin DM1, a microtubule inhibitor. When the conjugate binds to cancer cells, the microtubule inhibitor attached to the antibody is released. Emtansine, released by lysosomal enzymes, connects to the microtubules, leading to cell cycle retention and, as a result, apoptosis [143,144].

The Phase IIIb KAMILLA trial included patients with HER2+ MBC who had been previously treated with HER2-targeted therapy and chemotherapy with disease progression. In patients with baseline BrM, the median PFS was 5.5 months, and OS was 18.9 months. In patients with measurable BrM, the ORR was 21.4% [137]. Trastuzumab must maintain a higher level of HER2 expression in tumor cells compared to peripheral tissues to be effective. Loss of HER2, because of extended exposure to the drug, is a known resistance mechanism [144].

### 5.7. Lapatinib and Neratinib

An anilinoquinazoline derivative called lapatinib ditosylate monohydrate reversibly inhibits the HER1, HER2, and EGFR tyrosine kinases. It binds to the inactive form of EGFR, leading to its slower dissociation rate and resulting in its effect lasting longer at the target location than other EGFR tyrosine kinase inhibitors [145].

Neratinib is a second-generation, irreversible tyrosine kinase inhibitor against HER1, HER2, and HER4 and an anilinoquinoline derivative of pelitinib. It regulates cell proliferation and apoptosis by inhibiting the MAPK and AKT pathways [146].

In the NEfERT-T trial, neratinib-paclitaxel and trastuzumab-paclitaxel were assessed in women with previously untreated HER2+ MBC. One cohort received neratinib and paclitaxel, and the other, trastuzumab and the same dose of paclitaxel. The results showed that the incidence of CNS recurrences in the neratinib-paclitaxel group was lower (8.3%) than in the second group (17.3%). In the neratinib-paclitaxel group, 69.0% of patients had PFS events, and 65.8% in the trastuzumab-paclitaxel group. Another significant aspect was the delayed time of occurrence of BrM in the neratinib-paclitaxel group compared to the trastuzumab-paclitaxel one [147].

The NALA phase III trial showed that patients in the neratinib-capecitabine group required fewer intervention for BrM (22.8% vs. 29.2%), confirming the NEfERT-T trial finding. The most common AEs of any grade detected in this trial were diarrhea, nausea, palmar-plantar erythrodysesthesia syndrome, and vomiting [104].

The efficiency of neratinib was also evaluated in the ExteNET trial. Women with stage 1–3C HER2+ primary BC who completed standard locoregional treatment, neoadjuvant or adjuvant chemotherapy, and trastuzumab treatment within 2 years were divided into two classes, receiving either neratinib or placebo for 1 year. The number of CNS events in the HR+/1-year population was lower in the neratinib group (4 in 670 patients) than in the placebo group (12 in 664 patients), as well as the incidence of CNS recurrences. The 8-year OS in patients with residual invasive disease after neoadjuvant therapy receiving neratinib was 9.1%. Those results suggest that this drug may be an effective treatment for controlling CNS events (Table 3) [102].

## 6. Metastases to the Brain—New Therapeutic Methods

### 6.1. Trastuzumab Deruxtecan (T-DXd)

The antibody–drug combination, trastuzumab deruxtecan, is formed by a humanized monoclonal antibody that targets HER2 selectively, a cleavable tetrapeptide-based linker, and a strong topoisomerase I inhibitor. Regardless of target expression, trastuzumab deruxtecan may have a strong lethal effect on nearby tumor cells due to its released payload, which readily crosses the cell membrane, in contrast to trastuzumab emtansine. Furthermore, the short half-life of the delivered payload is intended to reduce systemic exposure [148]. It was approved for use on 20 December 2019 by the FDA for treating adults with unresectable or metastatic HER2+ BC who have received two or more prior anti-HER2-based regimens [93].

In the DEBBRAH phase II study, patients with stable, untreated, or progressing BrM or leptomeningeal carcinomas participated. Cohort 1 consisted of patients with non-progressing BrM after local therapy, cohort 2 consisted of patients with asymptomatic untreated BrM, and cohort 3 consisted of patients with progressing BrM after local therapy. All the participants received 5.4 mg/ kg T-DXd intravenously once every 21 days. In cohort 1, the 16-week PFS was 87.5%. The intracranial objective response rate (ORR-IC) in cohort 2 was 50.0%, and in cohort 3, it was 44.4%. The AEs were mostly grade 3 or higher neutropenia, fatigue, nausea, or vomiting [149].

Another trial demonstrating the efficacy of trastuzumab deruxtecan for the treatment of active BrM in patients with HER2+ BC is TUXEDO-1. A total of 60% of participants had BrM after previous local therapy, and 60% had received T-DM1. The ORR-IC by response assessment in neuro-oncology brain metastases (RANO-BM) was 73.3% of the ITT population. The median PFS came out to 14 months. The median OS was not reached. The leading AEs were grade 1/2 anemia, neutropenia, fatigue, and nausea [150].

The efficiency of T-DXd in the treatment of HER2+ BC BrM was performed using orthotopic patient-derived xenografts (PDX). Moreover, the effect of T-DXd was also assessed in a cohort study of 17 patients with stable or active BrM. The HER2+ PDX model results showed a tumor size reduction and prolonged animal survival. Moreover, it also reduced tumor size and prolonged survival in the T-DM1-resistant HER2+ BC BrM PDX model. The ORR-IC was 73%, in which 67% of patients had been previously untreated. The median overall PFS was not reached, while the 12-month overall PFS was 57.8% [151].

DESTINY-Breast12 is a phase 3b/4 study investigating T-DXd in patients with HER2+ BC also with BrM. A total of 59.7% of patients with baseline BrM had stable and previously treated brain metastases, and 40.3% of patients had active BrM, of which 36.8% had been untreated. For patients with baseline BrM, the 12-month overall PFS rate was 61.6%, and median PFS reached 17.3 months. The 12-month OS was 90.3% for patients with baseline BrM. In patients with stable BrM, the overall ORR was 49.7% compared to the group of active BrM, where the ORR was 54.7% [152].

The presence of brain metastases can lead to BBB disruption, forming the BTB, which has increased permeability. This process permits ADCs, such as T-DXd, to cross it and affect the tumor [153].

### 6.2. Tucatinib

Tucatinib is a tyrosine kinase inhibitor selective for HER2. It has been observed that it inhibits the phosphorylation of HER2 and its downstream effector AKT3 in HER2-overexpressed cell lines, according to in vitro studies [154]. It was approved in the United States on 17 April 2020, in combination with trastuzumab and capecitabine for treating patients with unresectable or metastatic HER2+ BC who have received one or more prior anti-HER2-based regimens [155].

A Phase I study of treatment with tucatinib and trastuzumab in patients with HER2+ BrM was performed on two cohorts: the first receiving tucatinib twice daily and the second once daily. In 83% of participants, disease progression was observed after previous brain radiation; in addition, the median number of treatments performed for MBC in both groups was two. The maximum tolerated dose (MTD) was 300 mg and 750 mg of tucatinib combined with trastuzumab. In the first group, the clinical benefit rate was 35%, and in the second, 53%. Moreover, in the first group, the intracranial response was shown in 12% of patients and 6% of patients. The median PFS among patients treated at MTD in the first cohort was 3.4 months, and 4.1 months in the second cohort. In the first group, the median OS was 9.1 months, and for the second group it was 17.9 months [156].

In the HER2CLIMB trial, 612 patients took part, including those with stable or active BrM, diagnosed with HER2+ MBC. They received tucatinib or placebo with trastuzumab and capecitabine. Results showed that for patients with active BrM at baseline, the median OS was 21.4 months in the tucatinib-trastuzumab group and 11.8 months in the placebo group. For patients with previously untreated BrM, the median OS was 6.3 months longer in the drug-combination group than in the placebo group. The median OS for patients with stable BrM at baseline receiving the tucatinib combination was 21.6 months, compared to 16.4 months shown by the placebo-combination group. For all patients with BrM, the median CNS-PFS was 5.7 months longer in the tucatinib-combination group than in the placebo-combination group (9.9 vs. 4.2 months), and in the first group, the risk of disease progression was reduced by 61.4%. The ORR-IC in the tucatinib-combination group was 47.3%, and in the placebo-combination group, it was 20.0% [157].

An interesting study was conducted by Amrell et al. evaluating the efficiency of tucatinib and irradiation of human cancer cell lines overexpressing HER2. A 200 kV X-ray machine irradiated cells at dosage rates ranging from 1.27 to 1.91 Gy/min. Results stated that this combinatorial treatment decreases metabolic activity, cell proliferation, and clonogenicity, and enhances apoptosis compared to irradiation alone. Moreover, alpelisib further reduces clonogenicity in the PI3KCA-mutated cell line. This leads to the conclusion that this combination may be more effective than a single treatment used for treating BC [158].

Frequently observed events, mostly grade 1 or 2, after using tucatinib are diarrhea, palmar-plantar erythrodysesthesia syndrome, nausea, fatigue, and vomiting [98].

Tucatinib, as a small TKI molecule, can more readily cross the BBB than larger molecules, allowing higher CNS concentration and more effective results [159].

### 6.3. Pyrotinib

Pyrotinib, an irreversible pan-ErbB TKI drug targeting EGFR, HER1, HER2, and HER4, was approved for use in combination with capecitabine in August 2018, in China [160]. Chen et al. evaluated the therapeutic effects of palbociclib, trastuzumab, pyrotinib, and fulvestrant in patients with HR+/HER2+ MBC with BrM. It included 15 patients with mostly 2–5 BrM, who had been treated previously. A total of 35.7% of patients experienced a CNS ORR, the median time to CNS progression was 8.5 months, and the median PFS was 10.6 months. The 12-month OS rate reached 43.3%. The most common AEs detected in this study were diarrhea, leukopenia, vomiting, and anemia. The results indicate that this drug combination is an effective treatment [161].

Another study was conducted to estimate the efficiency of pyrotinib in treating HER2+ MBC with BrM, this time with radiotherapy. A total of 40 patients took part, receiving either stereotactic radiotherapy or whole-brain radiotherapy in combination with pyrotinib (400 mg) and capecitabine (1000 mg/m^2^). As a result, the 1-year CNS PFS rate was 74.9%, while the median CNS PFS was 18 months. Moreover, the CNS ORR reached 85%. The median OS was not reached. The most frequent AE was diarrhea. Those statistics indicate that this treatment is associated with long intracranial survival benefits [162].

Ma et al. did a study investigating pyrotinib-based therapy in patients with HER2+ MBC. All the participants had pathologically confirmed HER2+ BC with measurable BrM, experienced progression after any CNS-targeted treatments, and did not receive pyrotinib treatment before. Patients were divided into three groups receiving the following: pyrotinib plus capecitabine (57.4%); pyrotinib plus nab-paclitaxel (27.9%); or pyrotinib plus vinorelbine (14.7%). Median PFS reached 8.6 months, and the median OS was 18 months. PFS in patients given pyrotinib and nab-paclitaxel was 10 months; for patients given pyrotinib and vinorelbine, it was 7.5 months. Patients receiving local treatment have better PFS and OS than those without it. Moreover, the nab-paclitaxel combined regimen secured the best peripheral ORR, making it 84.6%. Frequent AEs were grade 3 to 4 for diarrhea and neutropenia (Table 4). These results conclude that pyrotinib-based regimens are a safe treatment for HER2+ BrM. However, pyrotinib combined with nab-paclitaxel is the most effective and least toxic [163].

Pyrotinib, another small TKI molecule, has a low molecular mass, which facilitates its penetration through the BBB and allows it to act upon CNS metastases [164].

## 7. New Trials in the Treatment of HER2+ BC with Brain Metastases

In addition to approved new treatment methods, it is essential to add ongoing or completed trials with new drugs and/or new combinations of drugs that have not yet been approved. We decided to gather all the information in a table focusing on the drugs/procedures investigated and the purpose of these trials (Table 5).

We also wanted to mention studies enhancing BC detection. A study by Ma et al., published in 2020, investigates the application of surface-enhanced Raman spectroscopy (SERS) for BC detection. A porous silicon Bragg reflector (PSB) was developed to enhance the Raman signals of serum samples. The research primarily emphasizes the fabrication and characterization of PSB and its potential in BC detection [165]. Cheng et al. focused on enhancing BC detection through advanced spectroscopic techniques. This study uses a high-sensitivity thermally annealed silver nanoparticle/porous silicon Bragg mirror (AgNPs/PSB) composite substrate to increase the Raman spectroscopy (RS) signal of serum using the SERS technique. This technique offers more detailed spectral information compared to the traditional RS. The study processed raw spectral data using normalization, baseline correction, and polynomial smoothing. With a training duration of 4 milliseconds, the SERS-based model combined with the Support Vector Machine (SVM) method obtained 100% accuracy, sensitivity, specificity, and Area Under the Curve (AUC) value. These results highlight the possibility of combining advanced SERS substrates with machine learning techniques to diagnose BC quickly and precisely [166].

A phase II study conducted by Oberkampf et al. assessed the efficacy and safety of intrathecal (IT) trastuzumab in patients with HER+ BC who developed leptomeningeal metastases (LM). Weekly IT doses of 150 mg trastuzumab were administered to the patients. Nineteen patients were enrolled, of whom 84% had concomitant BrM. At 8 weeks, the median LM-related PFS was 5.9 months, the median OS was 7.9 months, and 74% of patients experienced no neurological progression. The treatment was well tolerated; no toxicity above grade 3 was reported [167].

Another interesting study used a mouse model to assess the efficacy of a single intrathecal administration of the adeno-associated virus serotype 9 (AAV9) vector encoding trastuzumab. Compared to controls, a significantly increased median survival in mice receiving AAV9.trastuzumab was observed. Moreover, the reduced development of brain tumors was noted. Furthermore, higher doses of AAV9.trastuzumab resulted in smaller tumor volumes. This study demonstrated that intrathecal delivery of trastuzumab via the AAV9 vector may offer a novel therapeutic approach for HER+ BC BrM [168].

Researchers developed mucic acid-based polymeric nanoparticles designed to cross the BBB via transferrin receptor (TfR)-mediated transcytosis. These nanoparticles were engineered to carry camptothecin (a potent chemotherapeutic agent) and trastuzumab. The growth of brain tumors was significantly inhibited in mice treated with TfR-targeted nanoparticles containing camptothecin and trastuzumab, compared to mice given free drug combinations or nanoparticles containing camptothecin or trastuzumab alone. The nanoparticles successfully traversed the BBB. This demonstrates the potential of TfR-targeted nanoparticles for delivering combination therapies across the BBB in the treatment of HER+ BC BrM [169].

Sevieri et al. invented a novel strategy for delivering trastuzumab across the BBB. A ferritin-based nanoconjugate was developed because ferritin interacts with transferrin receptors, which are highly expressed on the BBB. As a result, the ferritin-trastuzumab nanoconjugate demonstrated improved penetration of the BBB. Furthermore, an increased accumulation of trastuzumab was found in BrM sites. Compared to controls, treatment with the nanoconjugates significantly inhibited the growth of BrM in preclinical models. By reducing trastuzumab’s distribution to non-brain regions, the targeted administration method may decrease the risk of adverse effects. This study presents a promising approach to overcoming the challenges of delivering large therapeutic molecules across the BBB [170].

## 8. Conclusions

HER2+ BC is often associated with poor prognosis and can lead to BrM. Standard treatment options for HER2+ BC include trastuzumab, pertuzumab, and margetuximab. The first-line treatment for HER2+ MBC is trastuzumab plus pertuzumab in combination with taxane chemotherapy. However, HER2+ BC with BrM is treated with surgery, whole brain radiotherapy, stereotactic radiosurgery, and chemotherapy, as well as earlier mentioned trastuzumab, pertuzumab, trastuzumab emtansine, lapatinib, and neratinib.

Whole-brain radiotherapy is often used as a palliative treatment. Long-term side effects include radiation necrosis. Radiation necrosis is also often an adverse effect of stereotactic radiosurgery. In comparison to WBRT, stereotactic surgery minimizes harm to nearby tissues. Due to disruption of the BBB caused by prior treatment, trastuzumab is an effective treatment for BrM. Pertuzumab is often combined with trastuzumab for the treatment of HER2+ MBC. T-DM1 leads to cancer cell apoptosis, elongating the OS. Neratinib and lapatinib are both targeted tyrosine kinase inhibitors. However, neratinib, in comparison to lapatinib, provides an irreversible inhibition. Trastuzumab deruxtecan, compared to trastuzumab emtansine, may have a strong lethal effect due to its released payload, readily crossing the cell membrane. Tucatinib is approved for use in combination with trastuzumab and capecitabine, showing an elongated median OS in studies with patients with previously untreated BrM. The most frequent AEs linked to pyrotinib are diarrhea, leukopenia, vomiting, and anemia.

Various ongoing trials are being conducted to evaluate the efficiency of new drugs or new drug combinations for the treatment of HER2+ BC BrM. Many of them are focused on finding the best combination of already approved drugs, such as trastuzumab or pyrotinib, while some focus on finding new ones. HER2+ BC BrM will continue to be a major problem, forcing the development of modern solutions.

## Figures and Tables

**Table 1:** Molecular subtypes of BC.

Subtype	Luminal A	Luminal B	HER2-Enriched	Basal-Like/TNBC
IHC Phenotype	ER+	ER+	ER−	ER−
	HER2−	HER2+ and/or	HER2+	PR−
				HER2−
	PR ≥ 20%	And/or PR < 20%	PR−	
	Low Ki-67 < 20%	High Ki-67 ≥ 20%		

Abbreviations: IHC—immunohistochemistry, ER—estrogen receptor, PR—progesterone receptor, HER2—human epidermal growth factor receptor 2, TNBC—triple negative breast cancer, Ki-67 proliferation index—the percentage of positively stained, dividing tumor cells among all the malignant cells.

**Table 2:** Clinical trials for HER2+ breast cancers.

Trial	Phase	n	Type of Patients	Regimen	OS	PFS	DoR	ORR	iDFS
CLEOPATRA (NCT00567190)	III	808	HER2+ MBC, ≤1 prior HT therapy	Pertuzumab + trastuzumab + docetaxel vs. placebo + trastuzumab + docetaxel	57.1 months vs. 40.8 months (end-of-study)	18.5 months vs. 12.4 months	87.6 weeks vs. 54.1 weeks	80.2% vs. 69.3% CR 5.5% vs. 4.2% PR 74.6% vs. 65.2%	-
APHINITY (NCT01358877)	III	4804	Operable HER2+ primary breast cancer	Adjuvant therapy pertuzumab + trastuzumab + chemotherapy vs. placebo + trastuzumab + chemotherapy	92.7% vs. 92.0% (*p* = 0.078)	-	-	-	86.1% vs. 81.2%
PEONY (NCT02586025)	III	329	Early-stage or locally advanced HER2+	Adjuvant therapy pertuzumab + trastuzumab + chemotherapy vs. placebo + trastuzumab + chemotherapy	39.3 months vs. 21.8 months	EFS 84.8% vs. 73.7% DFS 86% vs. 75%	-	Breast pathologic CR 39.3% vs. 21.8% objective response during cycles 1–4 88.6% vs. 78.2% CR during cycles 1–4 11.0% vs. 10.0% PR during cycles 1–4 77.6% vs. 68.2%	-
SOPHIA (NCT02492711)	III	624	HER2+ MBC who have received prior anti-HER2 therapies and require systemic treatment	Margetuximab + chemotherapy vs. trastuzumab + chemotherapy	21.6 months vs. 21.9 months (*p* = 6204)	5.8 months vs. 4.9 months	-	CR 2.7% vs. 1.5% PR 19.5% vs. 14.5%	-
TAnDEM	III	207	HER2/hormone receptor–copositive MBC Previous treatment with tamoxifen or anastrozole (up to 4 weeks before assignment)—permitted. Prior chemotherapy for MBC within 6 months—not permitted.	Trastuzumab + anastrozole vs. anastrozole alone	28.5 months vs. 23.9 months (*p* = 0.325)	4.8 vs. 2.4 months	9.5 months vs. 10.0 months	PR 20.3% vs. 6.8%	-
eLEcTRA	III	370	HER2 and HR-positive MBC or LABC, no prior treatment	Letrozole alone vs. letrozole + trastuzumab	-	25% vs. 40% TTP 3.3 months vs. 14.1 months	12.2 months vs. 11.4 months	13% vs. 27% (*p* = 0.3124)	-
SYSUCC-002 (NCT01950182	III	392	ER + and/or PR + HER2+ MBC	Trastuzumab + endocrine therapy vs. trastuzumab + chemotherapy	-	19.2 months vs. 14.8 months	-	-	-
PERTAIN (NCT01491737)	II	258	HR+/HER2+ locally ABC or MBC	Pertuzumab + trastuzumab + AI +/− chemotherapy vs. trastuzumab + AI +/− chemotherapy	60.16 months vs. 57.17 months (final analysis)	18.89 months vs. 15.8 months (primary analysis) 20.63 months vs. 15.80 months (final analysis)	27.4 months vs. 16.36 months	Overall response rate 63.3% vs. 55.7% CR 7.3% vs. 0.9% PR 56% vs. 54.7%	-
ALTERNATIVE (NCT01160211)	III	369	HR+/HER2+ MBC, prior trastuzumab and ET	Lapatinib + trastuzumab + AI vs. lapatinib + AI vs. trastuzumab + AI	Overall survival events 75% vs. 63.3% vs. 67.5%	Lapatinib + trastuzumab + AI vs. trastuzumab + AI 11 months vs. 5.6 months	14 months vs. 11.1 months vs. 8.4 months	Overall response rate 31.7% vs. 18.6% vs. 13.7%	-
EMILIA (NCT00829166)	III	991	HER+ locally ABC or MBC, prior trastuzumab therapy	Trastuzumab emtansine vs. capecitabine + lapatinib	29.9 months vs. 25.9 months (final analysis)	9.6 months vs. 6.4 months	12.6 months vs. 6.5 months	-	-
TH3RESA (NCT01419197)	III	602	HER+ locally ABC or MBC, ≥2 prior regimens of HER2 directed therapy	Trastuzumab emtansine vs. treatment of physician’s choice	22.7 months vs. 15.8 months (final analysis)	6.2 vs. 3.3	Duration of the objective response 9.7 months (6.60 to 10.51) vs. NA (2.4 to NA) months	31.3% vs. 8.6%	-
KATHERINE (NCT01772472)	III	1486	HER2+ primary breast cancer, residual tumor presents pathologically in the breast or axillary lymph nodes following preoperative therapy	Adjuvant therapy trastuzumab emtansine vs. trastuzumab	-	-	-	-	88.3% vs. 77.0%
DESTINY-Breast03 trial (NCT03529110)	III	524	HER2+ unresectable and/or MBC, prior trastuzumab and taxane treatment	T-DXd vs. T-DM1	18.5 months to NA vs. 6.8 months	25.1 months vs. 7.2 months	20.3 months to NA vs. 12.6 months to NA	79.7% vs. 34.2%	-
DESTINY-Breast04 (NCT03734029)	III	557	HER2-low unresectable and/or MBC	T-DXd vs. physician’s choice standard treatment	Cohort of participants with HER2-low BC 23.9 months vs. 17.5 months	HR+ cohort 10.1 months vs. 5.4 months regardless of HR status 9.9 months vs. 5.1 months	Cohort of participants with HER2-low BC 10.7 months vs. 6.8 months	Cohort of participants with HER2-low BC CR 3.6% vs. 0.6% PR 49.5% vs. 16.0%	-
HER2CLIMB (NCT02614794)	II	612	HER2+ unresectable locally ABC or MBC	Tucatinib + capecitabine + trastuzumab vs. placebo + capecitabine + trastuzumab	21.9 months vs. 17.4 months	7.8 months vs. 5.6 months	8.3 months vs. 6.3 months	40.7% vs. 23.4%	
NALA (NCT01808573)	III	621	HER2+ MBC, ≥2 prior regimens of HER2 directed therapy in metastatic setting	Neratinib + capecitabine vs. lapatinib + capecitabine	24 months vs. 22.2 months	8.8 months vs. 6.6 months	8.54 months vs. 5.55 months	32.8% vs. 26.7%	
PHOEBE (NCT03080805)	III	240	HER2+ MBC patients, prior taxane and trastuzumab therapy	Pyrotinib + capecitabine vs. lapatinib + capecitabine	-	12.5 months vs. 6.8 months	-	-	-

Abbreviations: IHC—immunohistochemistry; ER—estrogen receptor; PR—progesterone receptor; HER2—human epidermal growth factor receptor 2; OS—overall survival; PFS—progression-free survival; DoR—duration of response; ORR—objective response rate; CR—complete response; PR—partial response; iDFS—invasive disease-free-survival; AI—aromatase inhibitor; HT—hormonal therapy; DXd—trastuzumab deruxtecan; T-DM1—trastuzumab emtansine; MBC—metastatic breast cancer; ABC—advanced breast cancer; BC—breast cancer.

**Table 3:** Current treatment for HER2+ breast cancer with BrM.

Treatment Type	Number of Lesions	Dose Management	Adverse Effects
WBRT	5–10	30 Gy in 10 fractions/20 Gy in 5 fractions	Memory disturbance, difficulty with complex problem-solving, ataxia, urinary incontinence, radiation necrosis
SRS	Max. 4	24 Gy for max. 2 cm diameter, 18 Gy for 2.1–3 cm diameter, 15 Gy for 3.1–4 cm diameter	Radiation necrosis
Trastuzumab		High-dose 6 mg/kg weekly	Mainly unspecified pain, asthenia, nasopharyngitis, rash, dyspepsia, paresthesia, infections
Pertuzumab		Initial dose of 840 mg, maintenance dose of 420 mg every 3 weeks	Diarrhea, fatigue, nausea, vomiting, constipation, dizziness, headache, insomnia
Trastuzumab emtansine		3.6 mg/kg every 3 weeks	Thrombocytopenia, increased aspartate aminotransferase levels, anemia
Lapatinib and neratinib		Lapatinib: 1.250 mg daily Neratinib: 240 mg daily	Diarrhea, nausea, palmar-plantar erythrodysesthesia syndrome, vomiting

Abbreviations: WBRT—whole brain radiotherapy; SRS—stereotactic radiosurgery.

**Table 4:** New treatment for HER2+ breast cancer with BrM.

Treatment Type	Dose Management	Adverse Effects	Approval
Trastuzumab deruxtecan	5.4 mg/kg intravenously once every 21 days	Neutropenia, fatigue, nausea, anemia, vomiting	20 December 2019, by FDA
Tucatinib	300 mg orally twice daily	Diarrhea, palmar-plantar erythrodysesthesia syndrome, nausea, fatigue, vomiting	17 April 2020, in the US
Pyrotinib	400 mg once daily	Diarrhea, leukopenia, vomiting, and anemia	August 2018, in China

**Table 5:** New, ongoing, or completed trials for HER2+ breast cancer BrM.

Trail ID	Date of Start	Drugs/Procedure	Phase	Aim
NCT04639271	1 January 2021	Pyrotinib, Trastuzumab, and Abraxane combined	Phase 2	To evaluate the efficacy and safety of pyrotinib combined with trastuzumab and abraxane in HER2+ MBC with BrM
NCT04334330	4 December 2020	Palbociclib, Trastuzumab, Pyrotinib, Fulvestrant combined	Phase 2	To evaluate the efficacy of the combination of palbociclib, trastuzumab, and pyrotinib with fulvestrant in ER/PR positive and HER2+ BC patients with BrM
NCT06253871	25 March 2024	IAM1363	Phase 1	To evaluate the safety and preliminary efficacy of IAM1363 in patients with advanced cancers that harbor HER2 alterations.
NCT03696030	31 August 2018	Chimeric Antigen Receptor T-Cell therapy	Phase 1	To evaluate the side effects and best dose of HER2-CAR T cells in the treatment of patients with recurrent BrM
NCT04348747	19 December 2022	Anti-HER2/HER3 Dendritic Cell Vaccine, Pembrolizumab combined	Phase 2	To evaluate the efficiency of dendritic cell vaccines against HER2/HER3 and pembrolizumab in treating triple-negative BC or HER2+ BC with BrM

Abbreviations: MBC—metastatic breast cancer, ER—estrogen receptor, PR—progesterone receptor, BC—breast cancer, HER2—human epidermal growth factor receptor 2, HER3—human epidermal growth factor receptor 3, HER2+—human epidermal growth factor receptor 2 positive, HER2 CAR T cells—HER2 chimeric antigen receptor T cells, BrM—brain metastases.
